# Exploring Oceans for Curative Compounds: Potential New Antimicrobial and Anti-Virulence Molecules against *Pseudomonas aeruginosa*

**DOI:** 10.3390/md21010009

**Published:** 2022-12-23

**Authors:** Daniela Coppola, Carmine Buonocore, Morgan Palisse, Pietro Tedesco, Donatella de Pascale

**Affiliations:** 1 Department of Ecosustainable Marine Biotechnology, Stazione Zoologica Anton Dohrn, Via Ammiraglio Ferdinando Acton 55, 80133 Naples, Italy; 2 Département des Sciences de la Vie et de la Terre, Université de Caen Normandie, Boulevard Maréchal Juin CS, CEDEX, 14032 Caen, France

**Keywords:** multidrug-resistant bacteria, *Pseudomonas aeruginosa*, marine compounds, anti-microbial activity, anti-virulence agents

## Abstract

Although several antibiotics are already widely used against a large number of pathogens, the discovery of new antimicrobial compounds with new mechanisms of action is critical today in order to overcome the spreading of antimicrobial resistance among pathogen bacteria. In this regard, marine organisms represent a potential source of a wide diversity of unique secondary metabolites produced as an adaptation strategy to survive in competitive and hostile environments. Among the multidrug-resistant Gram-negative bacteria, *Pseudomonas aeruginosa* is undoubtedly one of the most important species due to its high intrinsic resistance to different classes of antibiotics on the market and its ability to cause serious therapeutic problems. In the present review, we first discuss the general mechanisms involved in the antibiotic resistance of *P. aeruginosa*. Subsequently, we list the marine molecules identified up until now showing activity against *P. aeruginosa*, dividing them according to whether they act as antimicrobial or anti-virulence compounds.

## 1. Introduction

The emergence and rapid global dissemination of antibiotic resistance in pathogenic bacteria represent one of the greatest challenges in clinical practice that needs coordinated approaches at regional, national, and international levels [1,2]. 

The increasing number of these pathogens is closely correlated with nosocomial infections worldwide, which account for 7 and 10% in developed and developing countries, respectively [3], resulting in significant implications on healthcare systems and important global economic costs. It is estimated that by 2050 there will be approximately 10 million deaths a year due to multi-drug resistance (MDR) bacteria and an economic loss of around 100 trillion USD [4]. Moreover, even more alarming is the increasing number of infections caused by pathogens resistant to many (if not all) antibiotics currently available today. Due to the growing gap between clinical need and the limited number of new drugs in development, the existence of these MDR bacteria could lead back to a pre-antibiotic era, leading to devastating consequences to the health of patients [2]. This problem is even more severe for Gram-negative bacteria, as highlighted by the World Health Organization reports, which underline the pressing need for new antibiotics to treat these bacterial infections [5]. One of the most important species among MDR Gram-negative bacteria is *Pseudomonas aeruginosa*, a member of γ –proteobacteria, due to its high intrinsic resistance to several classes of antibiotics and capability to cause serious therapeutic problems, with significant levels of morbidity and mortality [6,7]. According to the Infectious Diseases Society of America, *P. aeruginosa* is part of a faction of antibiotic-resistant bacteria called ‘ESKAPE’ (*Enterococcus faecium*, *Staphylococcus aureus*, *Klebsiella pneumoniae*, *Acinetobacter baumannii*, *P. aeruginosa*, and *Enterobacter* spp.), characterized by different drug resistance mechanisms that represent new paradigms in pathogenesis, transmission, and resistance [8]. 

Therefore, it is urgent to identify novel antibiotics with new chemical structures and other approaches to combat MDR Gram-negative bacteria. To date, the antibiotics commonly used on the market have been isolated from terrestrial organisms or derived synthetically from fermentation products. On the contrary, marine organisms may lead to the discovery of new and unique structures when compared to their terrestrial counterparts. In fact, the marine habitats represent the largest ecosystem on Earth still underexplored. They are characterized by extremely variable and hostile physico-chemical parameters, including low temperature, limited access to light, high salinity, and high pressure, which push marine organisms to produce an extraordinary secondary metabolites diversity, with unmatched structures and excellent biological activities (e.g., antimicrobials), to cope with such extreme conditions.

To fight MDR bacteria, it is also imperative to understand the resistance mechanisms that pathogens have developed and their contribution to bacterial virulence. In the present review, we first discuss general mechanisms involved in *P. aeruginosa* antibiotic resistance. Subsequently, we emphasize the critical need to identify new natural antibiotics and/or anti-virulence agents, focusing the attention on the molecules of marine origin identified up until now, showing activity against *P. aeruginosa*.

## 2. *Pseudomonas aeruginosa*

*P. aeruginosa* was isolated for the first time in 1882 by Gessard and identified as a pathogenic strain in 1890 by Charrin [9]. Similar to other bacteria of the *Pseudomonas* genus, it is well known for its considerable metabolic versatility and ubiquitous distribution. *P. aeruginosa* is usually aerobic, but it also can grow in an anaerobic condition if nitrate, citrate, and arginine are present [10]. Moreover, it is able to colonize a wide range of environmental niches [11], including terrestrial and aquatic habitats, as well as the surface of animate (insects, plants, animals, and humans) and inanimate (generally hospital environment, such as distilled water, disinfectants, sinks, medical devices, and equipment) hosts. In fact, *P. aeruginosa* is the most frequent colonizer of medical devices (e.g., catheters, nebulizers, humidifiers), causing nosocomial infections, such as ventilator-associated pneumonia, meningoencephalitis, and sepsis [12].

This bacterium is the major opportunistic pathogen for humans, typically affecting immunocompromised patients, giving rise to airway and urinary tract infections, bloodstream infections, burn injury infections, hot-tub dermatitis, and outer ear infections (known as swimmer’s ear). 

*P. aeruginosa* commonly infects the respiratory tract of Cystic Fibrosis (CF) patients [13], resulting in an accelerated decline of pulmonary function. It colonizes approximately 70% of adult CF patients, representing the most common pathogen isolated from these infections [14,15]. CF is a monogenic disease caused by a mutation on chromosome 7 in the CF transmembrane conductance regulator (CFTR) gene. This mutation affects the chloride channel inducing the formation of a thicker and dry mucus layer on the lung wall, increasing the viscosity due to hyposecretion and hyperabsorption of electrolytes and water in the airways [16]. This alteration causes problems in antimicrobial immunity, creating a perfect environment for infection and growth of *P. aeruginosa* [17].

**Figure 1:**
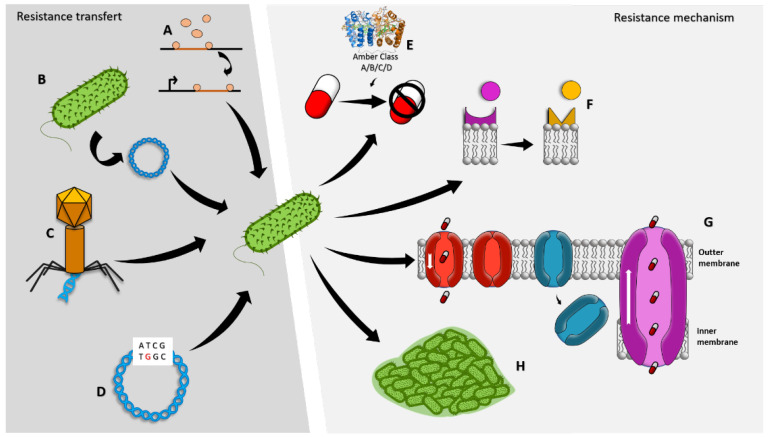
Schematic overview of acquired resistance and resistance mechanisms. Gene transfer via transposons (A), plasmids (B), bacteriophages (C); genome mutations (D); drug inactivation or alteration (E); modification of drug binding site (F); changes in cell permeability (G); biofilm production (H).

The treatment of infections caused by this pathogen is particularly problematic because of its natural and acquired resistance to multiple antibiotics, including aminoglycosides, quinolones, and β-lactams [18,19]. It is interesting that *P. aeruginosa* evolved the ability to find new ways to resist several compounds while simultaneously developing strategies for exchanging genetic materials, enabling other bacteria to also become drug-resistant (Figure 1) [20]. Its intrinsic resistance is commonly due to synergistic factors, including the impermeability of the outer membrane in Gram-negative bacteria, and the resistance developed through mutations in the genome or acquired from other organisms via plasmids, transposons, or bacteriophages [21]. Moreover, the pathogen can survive and adapt to several environments thanks to the signaling pathway that mediates antibiotic resistance, cell permeability, and the ability to form biofilm, as well as the production of different virulence factors such as cell-associated determinants (e.g., lipopolysaccharide, pili, and flagellum) and secreted molecules (e.g., extracellular polysaccharides, exotoxins, pigments, and proteases) [15,22,23]. These multiple resistance mechanisms represent potential drug targets that could guide the discovery of novel antibiotic adjuvants (i.e., compounds which do not directly kill bacteria but enhance antibiotic activity). Their action can block resistance, enhance the intracellular antibiotic accumulation and complementary bactericidal mechanisms, inhibit signaling and regulatory pathways, or boost the host response to bacterial infection [24,25].

## 3. Antimicrobial Resistance Mechanisms of *Pseudomonas aeruginosa*

To establish an infection, *P. aeruginosa* has developed many drug resistance mechanisms, including biofilm formation, modification of drug binding sites/targets, drug inactivation/alteration, and changes in cell permeability (Figure 1) [26].

### 3.1. Biofilm Production

Biofilm consists of a consortium of microorganisms that can live as a thin and slimy layer on biotic or abiotic surfaces or form aggregates without adhering to a surface, as seen in *P. aeruginosa*, *S. aureus,* and some other bacteria. It is established in a matrix of extracellular polymeric substances composed mainly of polysaccharides, proteins, lipids, and extracellular DNA (eDNA) [27]. The biofilm can represent a physical barrier or alter the chemical microenvironment (e.g., low O_2_, low pH, high CO_2_, and low water availability) to slow the diffusion and attenuate the action of antibiotics. Moreover, it allows the bacteria to resist adverse environmental conditions (e.g., starvation, desiccation) and makes them capable of causing a wide range of chronic infections [25,26,28].

The involvement of biofilm in drug resistance was highlighted by Ciofu and collaborators showing that the mucoid nature of biofilm was responsible for a high resistance toward Tobramycin in the pathogen *P. aeruginosa* [29]. Biofilm protects the pathogens increasing their tolerance against common antibiotics approximately 1000-fold [30]. Therefore, a high concentration of antibiotics would be required to treat biofilm infections, which cannot be utilized in vivo without causing toxicity [31], making the antibiotic treatment less adequate. In several cases, antibiotics (e.g., imipenem, colistin) can only reduce the biofilm without eliminating it. For this reason, the development of new antibiotic adjuvants that could eliminate the biofilm and that are effective when combined with antibiotics, represents a promising strategy for new therapies to prevent and treat bacterial infections. In this direction, it is interesting that Triclosan, a broad-spectrum antimicrobial [32], increased the efficacy of tobramycin against the pathogen by eradicating *P. aeruginosa* biofilm. The synergistic treatment of these two compounds caused a 100-fold reduction of viable persistent cells in 8 h and complete eradication by 24 h [33]. However, other anti-biofilm agents used as potential antibiotic adjuvants and their mechanisms of action have been previously reported [28].

Biofilm formation in *P. aeruginosa* represents an integral part of infections and is governed mainly by exopolysaccharides Pel and Psl [34,35,36]. Psl is enables to start the biofilm production and the maturation with the development of cell–cell communication, while Pel enhances the biofilm growth thanks to the cell–cell interaction [37,38]. *P. aeruginosa* can also synthesize alginate, which plays a key role in biofilm formation. The protein composition of the *P. aeruginosa* biofilm matrix has also been described, including type IV pili, Cup fimbria, CdrA adhesins, LecAB lectins, and Fap amyloid fibers [39]. In addition, it has been shown that eDNA represents a fundamental component in the matrix of the *P. aeruginosa* biofilm [40,41], together with rhamnolipids which are involved in the formation of microcolonies [42].

The biofilm formation involves several regulatory mechanisms (reviewed in [43]), including intracellular and intercellular signaling via secondary messengers (e.g., cyclic diguanylate monophosphate (c-di-GMP)) and quorum sensing (QS) molecules (e.g., homoserine lactones, quinolone), which are involved in initiating the transition to sessile development. A high internal concentration of c-di-GMP induces the production of adhesins and extracellular matrix compounds, allowing biofilm formation. On the contrary, the decrease in c-diGMP level causes biofilm dispersal [44,45]. Moreover, the acyl homoserine lactone-based system Rhl regulates rhamnolipid production and the tolerance of *P. aeruginosa* biofilms to immune cells [42,46], whereas the quinolone-based QS system, called the *Pseudomonas* quinolone signal (PQS), controls the production of the eDNA matrix component positively [41].

The mechanisms involved in the biofilm-associated tolerance of *P. aeruginosa* against the different classes of antibiotics are reviewed by Ciofu and collaborators [39]. Beta-lactams have little anti-biofilm effect, mainly due to the slow growth of bacteria in biofilms [39]. On the contrary, fluoroquinolones are frequently used to treat biofilm infections because they show good anti-biofilm effects and tissue penetration [47,48]. However, the low oxygen level in biofilms appears to be the main aspect affecting the bactericidal effect of quinolones, together with adaptive responses, including SOS and the stringent response [49]. Several mechanisms appear to have an important role in the tolerance of *P. aeruginosa* biofilms toward aminoglycoside antibiotics, such as the interaction of the aminoglycosides with different components of the biofilm matrix (e.g., alginate, Pel, Psl, eDNA) and the expression of specific genes that confer aminoglycoside tolerance of *P. aeruginosa* biofilm [39] and reference within.

### 3.2. Drug Inactivation

One of the principal intrinsic resistance mechanisms of *P. aeruginosa,* allowing cells to overcome the effect of antibiotic molecules, is the production of enzymes that can degrade antibiotics’ chemical bonds susceptible to hydrolysis, such as amides or esters [50,51,52].

Aminoglycosides are a group of bactericidal antibiotics whose mechanism of action involves the inhibition of protein synthesis. Structurally, aminoglycosides contain an aminocyclitol ring linked to amino sugars by glycosidic bonds [53]. Resistance to aminoglycosides is based on a series of different mechanisms, and the antibiotic degradation by enzymes is one of the principals. *P. aeruginosa* has been shown to produce the three different classes of enzymes known to degrade aminoglycosides: aminoglycoside acetyl-transferase (AAC), aminoglycoside nucleotidyltransferase (ANT), and aminoglycoside phosphotransferase (APH) [53,54]. Each class of enzyme has a specific antibiotic selectivity and a different mechanism of action. AACs are responsible for the inactivation of tobramycin, netilmicin, gentamicin, kanamycin, and amikacin by transferring an acetyl group to an amino group of the target molecules [51]. APHs inactivate neomycin, streptomycin, and kanamycin, transferring a phosphoryl group to the 3′-hydroxyl of aminoglycosides [51,55,56]. ANTs confer resistance to gentamicin, amikacin, and tobramycin by transferring an adenylyl group to either the amino or hydroxyl group of the antibiotics [57,58]. 

*P. aeruginosa* strains have developed several strategies for resistance to β-lactam antibiotics. The most common is represented by the expression of hydrolytic β-lactamase, an enzyme able to break the amide bond of the β-lactam ring. These enzymes are divided into four classes (A to D) based on their amino acid sequence. *P. aeruginosa* strains produce a class C β-lactamase that hydrolyze the cephalosporins β-lactam ring through an active site serine [59]. Other isolates have shown the production of a class A enzyme (same mechanisms of the C class) with a high degree of resistance for many β–lactam, defined extended-spectrum-β-lactamases (ESBLs). ESBLs confer resistance to penicillin, cephalosporins, and aztreonam [60,61]. Moreover, some OXA-type ESBLs were isolated in *P. aeruginosa,* and their name refers to their oxacillin-hydrolyzing abilities [61]. The overexpression of antibiotic-inactivating enzymes due to their mutation is another mechanism of acquired resistance used in *P. aeruginosa* [62]. An example is given by clinical isolates of *P. aeruginosa* in which the overproduction of β-lactamase caused by mutations in a β-lactamase inducible gene *ampC* significantly increased the resistance to cephalosporins [59]. Furthermore, mutations that inactivate the *ampD* gene encode a cytosolic N-acetyl-anhydromuramyl-1-alanine amidase and act as a repressor of *ampC* expression, led to an overproduction of β-lactamase in *P. aeruginosa* [63].

### 3.3. Modification of Drug Binding Sites/Targets

Interference with antibacterial targets is a strategy commonly used by pathogenic resistant bacteria to avoid the antimicrobial action of antibiotics. It can happen by securing targets or modifying the target sites [62]. In particular, mutational modifications of the target sites is an important strategy to contribute to *P. aeruginosa* antibiotic resistance.

One of the well-studied examples is the modification of the target sites of quinolones. Mutations in genes encoding for DNA gyrase (*gyrA* and *gyrB*) and/or topoisomerase IV (*parC* and *parE*) in *P. aeruginosa* can reduce the binding affinity of encoded proteins to quinolones, which generally inhibit the replication of the bacterial DNA, resulting in reduced susceptibility of the pathogen to quinolones [64].

High levels of resistance of *P. aeruginosa* to aminoglycosides have been reported in the presence of ribosomal mutations, as the antibiotic inhibits protein translation by targeting the 30S ribosomal subunit [65]. Similarly, it has been shown that modifications of *P. aeruginosa* penicillin-binding proteins (PBP) increase resistance to pan-β-lactam antibiotics [66].

Furthermore, it has been shown that resistance to polymyxin in *P. aeruginosa* is related to the modification of the polymyxin-binding partner lipopolysaccharides (LPS) by the addition of 4-amino-L-arabinose (L-Ara4N) to the phosphate groups of the lipid A moiety of LPS [67]. In addition, mutations in the two-component regulatory systems of PhoPQ and PmrAB promoted this modification, resulting in increased resistance to polymyxin [68,69].

### 3.4. Changes in Cell Permeability

Most antibiotics used to fight *P. aeruginosa* need to penetrate the cell membrane to reach their targets. This is the case of aminoglycosides (inhibition of protein synthesis), but also quinolones (inhibition of DNA gyrase and DNA replication) and β-lactam (inhibition of peptidoglycan synthesis) [70,71,72]. *P. aeruginosa* possesses different mechanisms to counteract antibiotics internalizations. The principal mechanism is represented by the membrane itself. *P. aeruginosa* has low membrane permeability (12- to 100- fold lower than that of *Escherichia coli*) [73] due to the relatively low number of porins, β-barrel protein channels responsible for the import of a broad range of molecules [74].

The most abundant porine is OprF which is present in two forms, the highly-abundant two-domain closed channel and the single-domain open channel. The open conformation occurs only in <5% of the OprF protein population [75], and this low incidence contributes to the low permeability of *P. aeruginosa* [76]. OprF is also implicated in quorum sensing and biofilm formation [74]. It was reported that some clinical OprF deficient mutants of *P. aeruginosa* have shown increased antibiotic resistance, although its role in antibiotic resistance still needs to be completely unraveled [77].

The second major *P. aeruginosa* porin is OprD which is involved in the recognition and entrance of carbapenem antibiotics [78,79]. In addition, mutations causing a downregulation of oprD have been connected to carbapenem resistance in some isolates [80]. 

However, if an antibiotic succeeds to reach the cell cytoplasm, other resistance mechanisms are activated. Bacterial efflux pumps play a fundamental role in expelling compounds out of the cell and are classified into the five following families: resistance-nodulation-division (RND) family, major facilitator superfamily (MFS), ATP-binding cassette (ABC) superfamily, small multidrug resistance (SMR) family, and multidrug and toxic compound extrusion (MATE) family [81]. *P. aeruginosa* possesses many efflux pumps belonging to the RND family to counteract antibiotics [82]. These proteins comprise a cytoplasmic and periplasmic component, named multidrug efflux (Mex), and an outer membrane porine (Opr). Four of the twelve *Pseudomonas* RND family efflux pumps, specifically MexAB-OprM, MexCD-OprJ, MexEF-OprN, and MexXY-OprM, contribute to antibiotic resistance [83]. MexAB-OprM is responsible for the efflux of β-lactams and quinolones [84]. MexCD-OprJ is able to pump out β-lactams [85]. MexEF-OprN can extrude quinolones [86], while MexXY-OprM expels aminoglycosides [84,87]. It has been reported that many *P. aeruginosa* strains present in the clinical environment showed an overexpression of many RND, underlining the importance of these mechanisms for antibiotic resistance [88,89,90]. 

## 4. Marine Natural Products against *P. aeruginosa*

Although medical procedures have limited the development and spread of pathogens through the use of antibiotic therapies, this has led to a global increase in resistant populations. Among them, a troubling example is the treatment of *P. aeruginosa* infection, which is complicated by the intrinsic resistance of the pathogen to a wide variety of antimicrobials. Furthermore, it acquires novel resistance mechanisms through lateral gene transfer, which probably also led to the emergence of carbapenemase-expressing strains of *P. aeruginosa* [91]. For this reason, *P. aeruginosa* is in the “critical” category of the World Health Organisation’s (WHO) priority list of pathogens for which research and development of new antibiotics is urgent [92].

Taking into account the growing threat of antimicrobial resistance, it is imperative to focus research not only on new antimicrobials discovery, but also on the development of innovative therapeutic approaches against the pathogen. Currently, a promising strategy appears to be targeting the virulence factors to suppress its ability to inflict damage, helping to slow down pathogenic mechanisms and facilitate bacterial clearance [93,94]. Anti-virulence compounds are based on inhibiting bacterial virulence and interrupting colonization processes and bacterial infections, but do not affect bacterial growth. They can be given in combination with antibiotic treatment, thus reducing the selective pressure on bacteria and preventing the development of resistance to these antibiotics [95]. In particular, QS and biofilm are the most frequently targeted virulence systems in *P. aeruginosa*.

Bacterial biofilm production represents one of the most relevant virulence factors, which can make pathogens up to 1000-times more resistant than their planktonic form [96]. Nowadays, the percentage of biofilm-mediated MDR infections is very high and growing. For this reason, the research for new therapeutic agents capable of counteracting biofilms formation is increasingly urgent. Likewise, the QS system represents an attractive target for developing novel antimicrobial drugs [97], as it plays an important role in establishing successful *P. aeruginosa* infections. Compared to conventional antibiotics that kill or inhibit bacterial growth, QS inhibitors prevent QS-regulated pathogenic processes [98,99], including biofilm formation, but also virulence factor expression (such as protease, exotoxin A, pyocyanin, pili, and flagella), bacterial migration and secretion regulation, putting less selective pressure on bacteria, thus barely inducing drug resistance mutations [100,101]. 

Therefore, the development of new molecules directed toward virulence targets could increase the therapeutic arsenal available for treating MDR pathogens.

In this context, marine organisms represent potential sources of novel therapeutic agents as they produce a wide diversity of bioactive compounds with pharmacological applications. They are adapted to survive in complex communities and in competitive and hostile habitats, producing unique secondary metabolites in response to ecological pressures, including competition for space, predation, and tide changes. Among these, many antimicrobial and/or anti-virulence compounds have been reported, which inhibit or limit the development and growth of other competitive marine organisms or inhibit virulence factors preventing the colonization of microorganisms and representing a further important therapeutic approach against pathogens. 

For this reason, here we list natural bioactive metabolites of marine origin which may represent important compounds against *P. aeruginosa*, dividing them according to whether they act as antimicrobial (Table 1) or anti-virulence (Table 2) compounds.

### 4.1. Antimicrobials

#### 4.1.1. Peptides and Proteins

The strong activity of the proteins/peptides makes them good drugs for the treatment of several diseases, although their instability remains a major problem for the therapeutic use. For this reason, the effective delivery of these drugs to the body represents an important challenge. However, many efforts have been made by scientists to find effective proteins/peptide drug delivery, including the stabilization of proteins and peptides in delivery devices and/or the design of appropriate target-specific protein transporters, as reviewed by [179].

A significant type of marine compounds able to counteract *P. aeruginosa* is represented by the antimicrobial peptides (AMPs), a class of small peptides with broad-spectrum antimicrobial activity that are components of the innate immune response widespread in the life kingdoms [180].

Mussels represent a relevant source of marine AMPs active against *P. aeruginosa*. Recently, four myticalins with MIC ranging from 4 to 8 µM were isolated from marine mussels belonging to the *Mytilus* genus, but their mode of action is still unclear [102]. Peptides from mussels can also be a valid starting point for the design of derived AMPs. For example, Oh and collaborators designed a short AMP from the immune-related AMP myticusin-beta produced by the *Mytilus coruscus* mussel in response to infections. The derived AMP showed increased antimicrobial action in the ultrasensitive radial diffusion assay (URDA) against a broad spectrum of human pathogens, including *P. aeruginosa* [103]. Among AMP from mussels, the polypeptide cgUbiquitin isolated from the oyster *Crassostrea gigas* gill showed potent bacteriostatic activity against *P. aeruginosa*, with a minimal effective concentration (MEC) value of 0.6 µM by URDA [104]. Mussels are also able to produce proteins with antimicrobial activity. This is the case of the escapin, a broadly antimicrobial protein of 60 kDa isolated from the ink of the sea hare *Aplysia californica*, that can inhibit *P. aeruginosa* with a MIC value of 0.31 µg/mL [121].

Edible sea urchin also represents a source of AMPs. In fact, three AMPs with strong antimicrobial activity were isolated from the *Echinus esculentus* collected from sub-Antarctic waters. In particular, the molecules EeCentrocin 1 and EeCentrocin 2 showed MIC of 0.78 µM against *P. aeruginosa*, while the EeStrongylocin 2 showed a MIC value of 1.56 µM [105].

Among invertebrates, crabs could also be important for producing marine AMPs. In this regard, the AMP crustin was purified from the hemolymph of the blue crab *Portunus pelagicus,* and its antimicrobial activity against *P. aeruginosa* was assessed by resazurin, which showed a MIC value of 50 µg/mL [106]. In addition, other three AMPs with a peculiar eight cysteines motif were isolated for the first time from the red king crab *Paralithodes camtschaticus*. These compounds, named paralithocin 1-3, have shown weak antimicrobial activity towards *P. aeruginosa*, with MIC value > 100 µM [107].

Sphistin, a histone-derived 38-amino acid peptide isolated from the mud crab *Scylla paramamosain* and its truncated fragment Sph_12–38,_ exhibited broad anti-microbial activities [181,182]. A recent study demonstrated that low doses of AMPs Sphistin and Sph_12-38_ in combination with rifampicin and azithromycin have in vivo significant synergistic activity against *P. aeruginosa*, probably due to the binding of these AMPs with LPS of *P. aeruginosa* with the subsequent promotion of the rifampicin and azithromycin intracellular uptake [108].

All D-amino acid analogue of tachyplesin I (TPI), a 14-amino acid AMP isolated for the first time from *Tachypleus tridentatus* [109] (commonly known as horseshoe crab), was synthesized by Yu and collaborators and named TPAD. It retained the TPI anti-microbial activity against *P. aeruginosa* with MIC values of approximately 8 μg/mL, but it showed significantly improved stability against enzymatic degradation and decreased hemolytic activity, suggesting better therapeutic potential [183].

Among marine organisms, vertebrates also represent a valid source of AMPs effective against *P. aeruginosa*. For example, a novel AMP with high homology with the C-terminus of hemoglobin β-chain was extracted from the liver of the skipjack tuna *Katsuwonus pelamis*. This AMP, which the authors designated as Skipjack Hemoglobin β chain-related Antimicrobial Peptide (SHβAP), showed broad spectrum bacteriostatic activity with an MEC value of 19 µg/mL towards *P. aeruginosa* [110].

Epinecidin-1, a 20-amino-acid peptide identified by orange-spotted grouper (*Epinephelus coioides*), revealed an important role in protecting fish against Gram-positive and -negative bacteria destroying membranes, probably thus preventing or delaying the development of microbial resistance [184]. In particular, this peptide inhibited in vivo the growth of *P. aeruginosa* ATCCS19660 and MDR *P. aeruginosa* R strains, with MIC_90_ (90% growth inhibition) values of 50 and 3.12 μg/mL, respectively. Importantly, the survival rate of mice after epinecidin-1 treatment was significantly higher than that of untreated controls or mice treated with imipenem [111].

Strong antimicrobial activity against *P. aeruginosa* (MIC value of 0.52 µg/mL) was also shown by the piscidin-like AMP named TP-4, which was isolated from the teleost fish *Oreochromis niloticus* [112]. Teleosts also represent a source of synthetic AMPs. In fact, three peptides (oreoch 1–3) with broad spectrum antimicrobial activity were synthesized using as guide the DNA of *Oreochromis niloticus*, two of which exhibited moderate activity against *P. aeruginosa*, with MIC values of 35 and 6.67 µM [113].

Besides teleosts, a novel cathelicidin (Hc-CATH) was identified and characterized from the sea snake *Hydrophis cyanocinctus.* This molecule has potent and broad-spectrum antimicrobial activity, showing a MIC value of 5.17 µM towards *P aeruginosa* [114]. Moreover, an AMP from the epidermal mucus of the hagfish *Myxine glutinosa L.* was able to completely inhibit the growth of two *P. aeruginosa* strains with MBCs of 7 and 10 µg/mL [115].

Marine microorganisms also represent a valid source of AMPs able to counteract the growth of *P. aeruginosa*. Among fungi, a cyclic dipeptide (L-Tyr-L-Pro) able to inhibit *P. aeruginosa* PAO1, with a MIC value of 6.2 mg/mL, was isolated from *Penicillium chrysogenum* DXY-1; moreover, anti-QS activity was also demonstrated (see Section 4.2.1) [116]. Moreover, cyclodepsipeptide was isolated from the ascidian-derived fungus *Aspergillus clavatus* AS-107. This compound showed potent antimicrobial activity against *P. aeruginosa*, with a MIC value of 8.8 µM [117].

Among marine-derived bacteria, three gageostatins (A–C) with antimicrobial activity were isolated from a *Bacillus subtilis* strain. Gageostatins A and B showed moderate antibacterial activity towards *P. aeruginosa* with a MIC value of 16 µg/mL when tested individually. Interestingly, the activity increased when combined with a MIC value of 8 µg/mL [118]. Moreover, two cyclic AMPs, hololitaralin A and B, previously isolated from the marine-derived *Halobacillus litoralis* YS3016, were synthesized, and their biological activities were evaluated by disk diffusion assay (DDA). The synthetic compounds showed activity against *P. aeruginosa* with 24 and 17 mm diameters, at 50 µg/mL for hololitaralin A and B, respectively [119,120].

Dusane and collaborators evaluated the antimicrobial activity of the protein BL-DZ1 produced by the bacterium *B. licheniformis* D1 isolated from the surface of the green mussel *Perna viridis*. This compound inhibited the growth of *P. aeruginosa* PAO1 at a MIC value of 3.12 µg/mL [122].

Gageotetrins A−C are rare bioactive linear lipopeptides consisting of a Leu-rich di- or tetrapeptide backbone and a new 3-hydroxy fatty acid (HDDA) isolated from a marine-derived *Bacillus subtilis* strain that showed anti-microbial activities against *P. aeruginosa* with MIC values of 0.02-0.06 mM [123].

#### 4.1.2. Terpenes and Terpenoids

Terpenes represent the major class of secondary metabolites and are constituted of isoprene units linked in different ways. Terpenoids are a modified class of terpenes widespread in nature, with a huge range of structures and different biological activities (including mainly anticancer, anti-inflammatory, and anti-viral activities), that can be found in bacteria, fungi, plants, and several invertebrates [185].

Among marine terpenes, only the conidiogenone B isolated from the marine-derived endophytic fungus *P. chrysogenum* QEN-24S exhibited antimicrobial activity against *P. aeruginosa*, with a MIC value of 8 µg/mL [124]. Likewise, a new ophiobolin sesterterpenoid and three new farnesylated phthalide derivatives, farnesylemefuranones, together with two known ophiobolin analogues, were also isolated from the deep-sea-derived fungus *Aspergillus insuetus* SD-512, collected from cold seep sediments in the north-east of the South China Sea, at a depth 1331 m. They demonstrated good anti-microbial activities against *P. aeruginosa*, with MIC values between 8 and 16 μg/mL [125].

Marine terpenoids with antimicrobial activity towards *P. aeruginosa* were also found in eukaryotes. For example, two terpenoids with antibacterial activity against *P. aeruginosa* were purified from the marine seaweed *Dictyoata acutiloba*, showing MIC values of 0.9 and 0.89 µg/mL in the microdilution assay [126]. Moreover, a new antimicrobial terpenoid was isolated from the red sea soft coral *Sarcophyton trocheliophorum*. This compound gave 8 mm of inhibition halo at 1 mg/mL against *P. aeruginosa* in the DDA [127]. Finally, three terpenoids were isolated from the marine sponge *Axinella infundibuliformis*, of which one exhibited very strong activity against *P. aeruginosa*, giving 24 mm of a diameter of the halo of inhibition in the DDA [128].

#### 4.1.3. Polyketides

Another relevant class of bioactive secondary metabolites is represented by polyketides (PKs). These molecules are produced by condensing short-chain fatty acids, typically acetyl-coenzyme A (acetyl-CoA) and malonyl-CoA, by polyketide synthases [186].

Two novel aromatic PKs, stremycin A and B, belonging to the angucycline antibiotic class, were isolated and chemically characterized from the bacterium *Streptomyces pratensis* strain NA-ZhouS1. Both molecules showed broad spectrum antimicrobial activity, with a MIC value of 16 µg/mL against *P. aeruginosa* [129]. Similarly, four new aromatic PKs, penipyranicins A−C and isopyrenulin, were isolated together with the known compound kojic acid from *Penicillium* sp. RO-11, collected from the sediments of a hydrothermal spring in Saudi Arabia. These compounds showed antimicrobial activity against *P. aeruginosa*; in particular, penipyranicin C had the most potent antimicrobial activity with a MIC value of 1.4 μg/mL [130].

Mollemycin A, a glyco-hexadepsipeptide PK containing two piperazic acids, was isolated from a marine-derived *Streptomyces* sp. CMB-M0244. It showed broad growth inhibitory activity against Gram-positive and Gram-negative bacteria, including *P. aeruginosa* (IC_50_ value of 50 nM) [131]. Moreover, new polyene pyrone PKs were purified from a marine fungus, *Penicillium* sp. BB1122, collected from the Zhoushan coast by applying the “metal-stress” strategy, which refers to the use of different concentrations of particular heavy metals that can induce the expression of cryptic gene clusters, with the consequent production of new secondary metabolites. They displayed considerable antibiotic activity against *P. aeruginosa*, with MIC values of approximately 4 μg/mL [132].

Microorganisms associated with marine invertebrates, such as sponges and algae, are also a well-known source of natural products, including antimicrobial PKs. From the marine *Streptomyces* sp. strain HB202 isolated from the sponge *Halichondria panicea,* a new cytotoxic PK was purified and characterized. This PK, a benz[*a*]anthracene derivative, was named mayamycin and showed promising antimicrobial activities with an IC_50_ value of 2.5 μM against *P. aeruginosa* [133]. Two of the three new PKs, ketidocillinones B–C, extracted from an Antarctica sponge-derived fungus *Penicillium* sp. HDN151272 exhibited inhibitory activity against *P. aeruginosa*, with MIC values of 1.56 and 6.25 mg/mL, respectively [134].

Recently, two antimicrobial macrocyclic PKs were isolated from *Shewanella algae* MTCC 12715, a symbiotic bacterium of the red macroalga *Hypnea valentiae*. The molecules were chemically characterized as 14-(14b,14c-dimethylbutyl)-12-methoxy-18-oxo-11,15-dioxacyclododecan-8-yl 1-((50-hydroxyfuran-10-yl)oxy)benzoate (**1**), and 14-(sec-butyl)-12-methoxy-12-methyl-18-oxo-11,15-dioxacyclododecan-8-yl1-((50-hydroxyfuran-10-yl)oxy)benzoate (**2**). Compounds **1** and **2** showed antimicrobial activity against *P. aeruginosa* with an inhibition halo of 21 and 24 mm diameter in DDA and MIC values of 1.5 and 3 µg/mL, respectively [135].

Recently, three elansolid-type of PK spanned macrolides isobenzofuranyl and furopyranyl, were obtained from the marine *Bacillus amyloliquefaciens* MTCC 12716 isolated from the intertidal red alga *H. valentiae*. They showed high antimicrobial activities against MDR strains, including *P. aeruginosa*, with a MIC value less than 1.0 μg/mL for the most active compound. Interestingly, the positive antibiotics such as ampicillin and chloramphenicol had the MIC values more than 12.5 μg/mL against *P. aeruginosa* [136]. Similarly, macrobrevin analogues were isolated by bioactivity-guided purification from *B. amyloliquefaciens* MTCC 12713 associated with an intertidal red macroalga *Kappaphycus alvarezii* collected along the south-east coast of India [137]. These molecules identified as trihydroxy-decahydro-37-methyl-macrobrevin, hexahydro-macrobrevin, hexahydro-41- hydroxy-macrobrevin-31-acetate, and hexahydro-28-nor-methyl-5-methoxy-macrobrevin, showed considerable antibacterial activity against *P. aeruginosa* with a range of MIC values from 1.56 to 6.25 μg/mL. In comparison, the antibiotic chloramphenicol used as a positive control exhibited a MIC value of 12.5 μg/mL. In addition, three of these four purified compounds also showed a wider inhibition zone against *P. aeruginosa* (19–23 mm using 30 μg of each sample on disc) than the positive controls chloramphenicol and ampicillin (11 mm). In particular, the macrobrevin molecule encompassing hexahydro-41-hydroxy-macrobrevin-31-acetate functionality showed the highest antimicrobial activity compared to the others [137]. Moreover, from the same bacterium, 21-membered macrocyclic lactones, identified as difficidin analogues, were also isolated showing important antimicrobial properties [138]. They exhibited inhibition zones between 17 and 26 mm against *P. aeruginosa* (using 30 μg of each sample on disc) and MIC values of about 2–6 × 10^−3^ μM, compared to 11 mm (inhibitory zone) and 4.9 × 10^−2^ μM (MIC value) obtained with chloramphenicol [138].

The pentacyclic PK RF-3192C was purified from the fungus *Aspergillus niger* ASSB4 isolated from the marine red algae *Laurencia obtuse*. This compound revealed high activity against *P. aeruginosa* ATCC27853 in the DDA with 15 mm of inhibition zone [139]. Microketides A and B are new C-11 epimeric PKs purified from the gorgonian-derived fungus *Microsphaeropsis* sp. RA10-14 sampled in the South China Sea. They showed marked antimicrobial activity against *P. aeruginosa*, with MIC values of 0.19 and 1.56 μg/mL, respectively. In particular, microketide A exhibited the same MIC value of the positive control ciprofloxacin (0.19 μg/mL) [140].

#### 4.1.4. Alkaloids

Alkaloids represent another huge class of natural products with a wide range of biological activities mainly produced by plants but also found in microorganisms and marine organisms [187]. For instance, the alkaloids caerulomycin A and C, isolated from a marine-derived actinomycete *Actinoalloteichus cyanogriseus* WH1-2216, were active against *P. aeruginosa*, with MIC values of 21.8 μM and 38.6 μM, respectively [141]. Moreover, a potent antimicrobial alkaloid, crambescidin 800, isolated from the sponge *Clathria cervicornis,* showed a MIC value of 1 µg/mL against *P. aeruginosa* [142]. Bromoageliferin, a known alkaloid isolated from the sponge *Agelas dilatata,* showed significant growth inhibition of *P. aeruginosa*, with MICs of 8–32 μg/mL (11.45–45.83 µM); moreover, the inhibition of biofilm production was also observed (see Section 4.2.3) [143]. The cyclostellettamines also represent a group of strong bioactive alkaloids. Three different cyclostellettamines isolated from the marine sponge *Pachychalina* sp. showed high activity against *P. aeruginosa*, with MIC values depending on the size of their alkyl-chain size. Cyclostellettamine C showed a MIC value of 8.6 µg/mL against an antibiotic sensitive strain of *P. aeruginosa* and 18.8 µg/mL against two MDR *P. aeruginosa* strains. Similarly, cyclostellettamine E showed activity ranging from 9.4 to 18.8 µg/mL against sensitive and MDR *P. aeruginosa* strains. Finally, cyclostellettamine F exhibited strong activity towards MDR strains with MIC values ranging from 4.7 to 9.4 µg/mL [144]. The Red Sea sponge *Callyspongia siphonella* led to the discovery of two brominated oxindole alkaloids (**1** and **2**), which showed MIC values of 256 µg/mL against *P. aeruginosa* and considerable anti-biofilm activity (see Section 4.2.3) [145].

From the marine sponge *Dendrilla nigra*, three new alkaloids named denigrins A–C were isolated. The new molecules, characterized by a 3,4-diaryl pyrrole structure, possessed strong antimicrobial activities against a panel of human pathogen bacteria. In particular, the strains were able to inhibit the growth of *P. aeruginosa* with MIC values of 100, 25, and 12.5 µg/mL for A, B, and C forms, respectively [146]. The lack of the p-hydroxyphenyl group at C-2 of compound A may be responsible for the differences in the activities. In addition, Zhidkov and collaborators have recently synthesized new brominated fascaplysins, bis-indole alkaloids endowed with many biological activities originally isolated from the sponge. In particular, the 14-bromoreticulatine derivative showed selective activity against *P. aeruginosa* with a clearance zone of more than 35 mm at the concentration of 0.2 mg/disc in the Disk Diffusion Soft Agar Assay [188,189].

Beside sponges, other marine organisms are good sources of new antimicrobial compounds against *P. aeruginosa*. For example, from the arctic bryozoan *Tegella* cf. *spitzbergensis,* the compound ent-eusynstyelamide B, the enantiomer of the known brominated tryptophan metabolite eusynstyelamide, was isolated together with three new derivatives, eusynstyelamides D, E, and F. These molecules displayed antimicrobial activities with MIC values between 25 and 12.5 µg/mL [147].

#### 4.1.5. Miscellaneous

Beyond the four major classes of natural products described above (peptides/proteins, terpenes/terpenoids, PKs, and alkaloids), many other different marine compounds were able to inhibit *P. aeruginosa* in vitro assays.

On one side, from higher marine organisms, the flavone Velutin, extracted from the marine algae *Acanthophora spicifera*, revealed antimicrobial activity against *P. aeruginosa* in the DDA with an inhibition halo of 26.8 mm using 0.1 mg [148]. Moreover, the steroid Siphonocholin (Syph-1) isolated from the marine sponge *Siphonochalina siphonella* inhibited the growth of *P. aeruginosa* at the MIC value of 64 µg/mL. In addition, anti-biofilm and anti-QS activities were reported (see Section 4.2.4) [149]. Finally, a polybrominated diphenyl ether, 2-(20,40-dibromophenoxy)-3,5-dibromophenol, was isolated from the marine sponge *Dysidea granulosa* and showed a strong and broad-spectrum antibacterial activity, including an important activity against *P. aeruginosa* with a MIC value of 4 mg/L [150].

On the other side, among the metabolites produced by marine microorganisms, two diphenylether derivatives from a marine algae-derived fungus *Aspergillus versicolor* OUCMDZ-2738 were able to inhibit *P. aeruginosa* with MIC values lower than 18 µM [151]. In addition, ecteinamycin, a polyether antibiotic isolated from a marine-derived *Actinomadura* sp. collected from the ascidian *Ecteinascidia turbinate*, showed strong antibacterial activity against *P. aeruginosa* with a MIC value of 8.0 μg/mL [152]. Finally, from the red algae *H. valentiae* associated *B. amyloliquefaciens* MTCC 12716, three homologue members of the 24-membered macrocyclic lactone family were isolated and named bacvalactones 1–3. Each compound bears peculiar chemical functionalities and revealed potential inhibitory effects on several pathogens. Specifically, the three molecules were able to inhibit the growth of *P. aeruginosa* in vitro with MIC ≤ 3.0 μg/mL, lower than the MICs of standard antibiotic molecules [158].

Moreover, the genus *Bacillus* was revealed to be an important source of bioactive molecules against *P. aeruginosa*, such as the phthalate derivative di-(2-ethylhexyl) phthalate (DEHP) from a marine *B. subtilis* active at the MIC value of 8 µg/mL [153], the two novel glycolipids (ieodoglucomide C and ieodoglycolipid) isolated from *B. licheniformis* 09IDYM23, active at the MIC value of 0.01 and 0.03 µM, respectively [154], and the compound PAGI264 produced by *Bacillus* sp. REB264, able to inhibit the growth of *P. aeruginosa* in microdilution assay with a MIC of 15 µg/mL [155]. Moreover, macrolactines are a class of compounds mainly produced by *Bacillus* strains and show antimicrobial activity because of their unique chemical architecture. From the ethyl acetate extract of a marine *Bacillus* sp., isolated from sediment samples collected from Chuuk, Federated States of Micronesia and Ieodo, Republic of Korea’s southern reef, two new glycosylated macrolactins A1 and B1 were isolated and characterized. These molecules showed uncommon structural features and antimicrobial activity against *P. aeruginosa* with MIC values of 0.055 μM [156]. In addition, from Korean marine sediments, a new *Bacillus subtilis* strain was isolated producing new macrolactine derivatives. These new molecules, named gageomacrolactins 1–3, were characterized by the presence of sugar moieties and possessed antimicrobial activities against *P. aeruginosa* with MIC of 0.03 μM for compound **1** and 0.05 μM for compounds **2–3** [157].

A peculiar type of macrolactine was isolated from *Bacillus subtilis* MTCC 10403 isolated from brown seaweed *Anthophycus longifolius*. The molecule was defined as aryl-crowned macrolactine and displayed antimicrobial activities with siderophore-like mechanisms with a MIC < 13 μg/mL against *P. aeruginosa* [159].

### 4.2. Anti-Virulence Compounds

#### 4.2.1. Proteins and Peptides

Enzymatic disruption of the biofilm or the QS signal molecules is a promising therapeutic strategy for treating *P. aeruginosa* infections.

The alginate lyase (AlyP1400, 200 U/mL) purified from a marine bacterium of the genus *Pseudoalteromonas* was able to degrade around 90% of the alginate isolated from a mucoid *P. aeruginosa* clinical isolate CF27 and disrupted in vitro the biofilm grown in a dose-dependent manner. On the contrary, the heat-inactivated AlyP1400 lost those abilities. Moreover, the authors demonstrated that the synergy of the enzymatic activity of AlyP1400 with antibiotics reduced the CF27 biofilm biomass and enhanced the bactericidal activity of antibiotics, suggesting a potential therapeutic activity for the combinational use of alginate lyase and antibiotics to treat *P. aeruginosa* biofilm-related infections [160,161]. Interestingly, biofilm formation of *P. aeruginosa* PAO1 was remarkably reduced by 0.01 mg/mL of the extracellular alkaline protease produced by marine bacteria *Pseudoalteromonas* sp. 129-1, and almost entirely abolished with the concentration of 1 mg/ml. The purified protease also showed high tolerance to salt and organic solvents, indicating its promising prospect as an additive in laundry detergent and non-toxic anti-biofilm agents [162]. In addition, it has recently been identified an antibiofilm protein, Alterocin, secreted by the non-pigmented marine bacterium *Pseudoalteromonas* sp. strain 3J6 isolated from the Morbihan Gulf, Brittany, France, active against marine and terrestrial bacteria, including *P. aeruginosa* clinical strains [163].

An AdiC-like quorum quenching enzyme, YtnP, was cloned from a deep-sea probiotic bacterium, *Bacillus velezensis* (DH82 strain), isolated from the Western Pacific Yap trench and heterologously expressed in *E. coli* to investigate its applications on the improvement of hygiene problems caused by biofilm infection of *P. aeruginosa* in dental units. The results showed that YtnP was able to interrupt the QS of the pathogen by degrading the N-acyl homoserine lactones (AHL), thus inhibiting the *P. aeruginosa* EPS generation, biofilm formation, and virulence factors production (pyocyanin and rhamnolipid) significantly, and therefore, increasing the efficiency of antifouling against *P. aeruginosa* [164]. Similarly, the gene *ahaP*, encoding the enzyme AHL-acylase responsible for the quorum quenching activity against several synthetic AHLs, was identified in *Psychrobacter* sp. M9-54-1 isolated from the microbiota of holothurians. In vivo results showed that the heterologous expression of *ahaP* in *P. aeruginosa* PAO1 reduced the expression of the QS-controlled gene *lecA*, encoding for a cytotoxic galactophilic lectin and swarming motility protein, confirming its interference with the QS systems of the pathogen [165].

The Nesfactin lipopeptide, produced by marine bacteria *Nesterenkonia* sp. MSA31, was recently isolated and characterized by Kiran and collaborators [166]. The protease, esterase, lipase, and phospholipase activities, representing virulence phenotypes, were significantly reduced in both *P. aeruginosa* PAO1 and FSPA02 when treated with 50 µg/mL of Nesfactin. In addition, the production of the virulence factors pyocyanin, alginate, elastase, and rhamnolipid in PAO1 and FSPA02 was also reduced by 70%, 65%, 79%, and 75%, respectively, in the presence of 50 µg/mL of the lipopeptide. Finally, Nesfactin at 100 µg/mL disrupted 85–90% of the biofilm of both *P. aeruginosa* strains [166].

Cyclic dipeptides have been reported to exhibit different biological activities and are therefore considered promising building blocks of drug candidates [190,191]. As they contain endogenous amino acids, peptides generally exhibit high activity and low toxicity. However, they have a short half-life and low oral bioavailability. On the contrary, the advantage of using cyclic dipeptides over linear oligopeptides is their stability and oral bioavailability due to the lack of target bounds for exopeptidases.

A cyclic dipeptide, isolated from the marine fungus *P. chrysogenum* DXY-1 discovered from sediments surrounding the Taiwan Strait and identified as cyclo(L-Tyr-L-Pro), showed anti-QS activity against *P. aeruginosa* PA01. After treatment with this cyclic dipeptide at sub-MIC concentration (0.5 mg/mL), the production of pyocyanin and activities of proteases and elastase activity of *P. aeruginosa* PA01 were inhibited by 41%, 20%, and 32%, respectively. Furthermore, at the same concentration of cyclic dipeptide, a reduction of the biofilm of 48% and a decreased QS gene expression were observed [116]. It has also been shown that *P. chrysogenum* produces another compound with anti-QS activity, identified as tyrosol, which decreased QS-regulated pyocyanin production, elastase activity, and proteolytic activity by 63.3%, 57.8%, and 9.9%, respectively, and inhibited the biofilm formation in *P. aeruginosa* PA01, at a concentration of 0.5 mg/mL, without an effect on cell growth [167].

Moreover, a diketopiperazine (DKPs) identified as cyclo(Trp-Ser) was extracted from the marine bacterium *Rheinheimera aquimaris* QSI02 isolated from a dredge of the Yellow Sea. It showed anti-quorum sensing (anti-QS) activity, decreasing QS-regulated pyocyanin production, elastase activity, and biofilm formation in *P. aeruginosa* PA01 by 65%, 40%, and 59.9%, respectively, at a sub-MIC concentration (0.2 mg/mL) [168].

#### 4.2.2. Polyketides

Recently, Wang et al. investigated the ability of the PK cladodionen, produced by the marine fungus *Cladosporium* sp. Z148, to reduce the production of virulence factors in *P. aeruginosa* PAO1 [170]. The production of elastase, pyocyanin, and rhamnolipid was reduced by 36%, 24%, and 35%, respectively, when PAO1 was treated with 400 µM cladodionen. Moreover, this compound also reduced biofilm production by 52% at 400 µM. The authors related the reduction of these virulence factors to the reduction in the mRNA levels of their regulator systems: *las*, *rhl,* and *PQS* [170].

Zhang et al. isolated for the first time the PK equisetin from a marine fungus, *Fusarium* sp. Z10, assessing its anti-QS inhibitory activity against *P. aeruginosa* PAO1. This compound reduced the biofilm formation by 58% at 300 µM and the production of pyocyanin, rhamnolipid, and elastase by 60%, 56%, and 46% at 300 µM, respectively. In addition, equisetin also downregulated the expression of *las*, *rhl*, and *PQS* systems at 300 µM [171].

#### 4.2.3. Alkaloids

Bromoageliferin, a known alkaloid, was isolated from an organic extract of the sponge *Agelas dilatata* from the coastal area of the Yucatan Peninsula (Mexico) [143]. It showed significant activity against clinical strains of *P. aeruginosa*, inhibiting growth (see Section 4.1.4) and biofilm production of approximately 30–40% at concentrations of 8 or 16 mg/L (11.45 or 22.9 µM). Moreover, the in vivo efficacy was tested in the *Galleria mellonella* model of infection. The assay showed an increased survival time in larvae infected with the *P. aeruginosa* ATCC 27853 strain when treated with Bromoageliferin (2 mg/kg) [143].

In addition, LC-HRESIMS-assisted chemical investigation along with bioactivity-guided fractionation of the Red Sea sponge *Callyspongia siphonella* led to targeting two brominated oxindole alkaloids (**1** and **2**), not isolated from natural sources before, which showed considerable anti-biofilm activity in *P. aeruginosa* (49.32 + 1.18 % and 41.76 + 1.33% inhibition, respectively) [145].

#### 4.2.4. Miscellaneous

Other molecules not belonging to the classes mentioned above were also found to disrupt *P. auruginosa* QS systems and biofilm formation and virulence. From the extract of the seagrass *Halodule pinifolia,* 4-methoxybeanzoic acid (4-MBA) was isolated and evaluated for its potential. At the concentration of 62.5 μg/mL, it was able to prevent biofilm formation and also downregulate QS-mediated transcript and virulence factors [173].

Other low molecular weight molecules showed similar activities. Cyclo(-Leu-Pro) and 4-Hydroxyphenylacetamide (4-HPA) were isolated from the marine fungus *Pestalotiopsis sydowiana.* They were found to dramatically downregulate biofilm formation and virulence factors transcription at sub-optimal MIC concentration (125 μg/ml and 62.5 μg/ml, respectively) [169]. From a marine strain belonging to *Vibrio alginolyticus* species, tyramine and N-acetyltyramine were identified as responsible for reducing virulence factors of *P. aeruginosa*. Particularly, the production of pyoverdine, a siderophore linked to virulence, was reduced by 65% at the concentration of 1 mg/mL for both molecules [165].

The marine steroid Syph-1 isolated from the sponge *S. siphonella* was evaluated for biofilm and pellicle formation inhibition and anti-QS property against *P. aeruginosa*. At selected sub-MICs (>64 µg/mL), Syph-1 significantly decreased the production of QS regulated many virulence functions of PAO1 (elastase, total protease, pyocyanin, chitinase, exopolysaccharides, and swarming motility) as well as biofilm formation [149].

From the marine strain *Oceanobacillus* sp. XC22919, three molecules were identified as 2-methyl-N-(2′-phenylethyl) butyramide, 3-methyl-N-(2′-phenylethyl)-butyramide, and benzyl benzoate and evaluated for the ability to reduce virulence factors of *P. aeruginosa* PA01. Results showed that all three compounds, despite having no effect on the growth rate of the pathogen, were able to significantly decrease the pyocyanin production as well as the proteolytic and elastase effect at the concentration of 100 µg/mL. Furthermore, 2-methyl-N-(2′-phenylethyl) butyramide and benzyl benzoate were also able to inhibit biofilm formation of 50% and 37%, respectively, at the concentration of 100 µg/mL [172].

The lactone butenolide (5-octylfuran-2(5H)-one) was isolated from a marine *Streptomices* sp. and was known for its antifouling activities [192]. Yin and collaborators have then recently evaluated this compound for its antibiofilm activities. Interestingly, this molecule was able to inhibit biofilm formation and eradicate mature *P. aeruginosa* PA01 biofilm at the concentration of 800 µg/mL [174].

From a coral symbiont, a Gram-positive bacterium was isolated and identified as *Staphylococcus hominis*. This strain was selected for its ability to inhibit the biofilm formation of *C. violaceum.* From its extract, the compound responsible for the activity was identified as DL-homocysteine thiolacton, an analogue of acyl-homoserine lactones. Tested against *P. aeruginosa*, this molecule was able to inhibit biofilm formation at the concentration of 10 µg/mL. Furthermore, the expression analysis showed that DL-homocysteine thiolacton was able to downregulate many genes involved in QS regulation [175].

Balan and collaborators characterized a new glycolipid produced by a marine *Staphylococcus saprophyticus* SBPS-15 with anti-biofilm activity. This compound, staphylosan, showed 96% biofilm inhibition and 100% biofilm dislodging against *P. aeruginosa* BHKH 19 at 400 µg/mL [176]. Similarly, a glycolipid produced by a tropical marine strain *Serratia marcescens* was characterized. It showed anti-adhesive activity, inhibiting the attachment of *P. aerugnosa* PAO1 of 75% at 50 µg/mL. The authors also investigated the ability of this biosurfactant in removing the biofilm of *P. aerugnosa* PAO1 on both polystyrene plates and glass surfaces, achieving 62% and 71% of biofilm disruption at 50 µg/mL, respectively [177].

Kwan and collaborators investigated the quorum quenching activity of the lyngbyoic acid isolated from the marine cyanobacterium *Lyngbya majuscola*. This fatty acid reduced the production of the virulence factors pyocyanin and elastase in *P. aeruginosa* PAO1, inhibiting the expression of the genes required for their biosynthesis. Moreover, lyngbyoic acid directly inhibited in vitro purified elastase precursor LasB [178].

#### 4.2.5. β-Lactamases and Efflux Pump Inhibitors

As reported above (see paragraphs 3.2 and 3.4), the production of β-lactamases and the expression of efflux pumps belonging to the RND family are two important resistance mechanisms and, therefore, two potential targets to cope with AMR [193,194]. These molecules are supposed to work in combination with antibiotics, lowering their MICs. At the moment of writing, there is just one example of a marine-derived molecule with these activities against *P. aeruginosa,* 3,4-dibromopyrrole-2,5-dione, isolated from the marine pathogen *Pseudoalteromonas piscicida*. This molecule acts effectively as an Efflux Pump Inhibitor (EPI) by decreasing the MICs of several antibiotics up to 16-fold in *E. coli* strains overexpressing *P. aeruginosa* RND transporter MexAB-OprM/MexXY-OprM. Nowadays, EPIs and β-lactamase inhibitors discovery is principally done using chemical libraries looking for analogue molecules of already known inhibitors. This first result represents a fundamental finding, proving that marine habitat represents a valid source of new molecules and prompts the better exploitation of marine extracts to identify novel inhibitors.

## 5. Conclusions

To date, MDR bacterial infections are a severe global health problem [195], causing approximately 700,000 deaths each year worldwide. If the increase in antibiotic resistance is not reversed in the coming years, this could result in the deaths of 10 million people every year and a huge negative impact on the economy, with a reduction of about 1% of the gross domestic product (GDP) worldwide and a 5-7% loss in developing countries by 2050 [196].

Therefore, the need for new therapies to treat infections caused by MDR pathogens is indisputable. For this reason, growing attention has recently been given to the development of innovative projects for the design of new antimicrobial drugs, the identification of promising species, the isolation/characterization of compounds, and the evaluation of their safety, as well as the evaluation of synergistic effects between the components, are needed. Moreover, new strategies targeting microbial virulence factors or resistance mechanisms, are emerging as promising approaches to deal with the infections of MDR bacteria. For this purpose, oceans, thanks to their unique habitats, including high-pressure, high-salt, low-temperature, hypoxic, and oligotrophic ecological environments, provide marine organisms with unique metabolic strategies, considerably increasing the probability of new lead compounds discovery. Moreover, we have explored just 5% of the ocean, meaning its hidden treasure of new bioactive compounds is still available for human uses. For this reason, this review highlights the great potential of marine organisms for discovering new molecules to cope with the MDR threat.

## Figures and Tables

**Table 1:** List of marine antimicrobial compounds against *P. aeruginosa*.

Producer Organism	Type of Organism	Compound	Class ^a^	Activity	Assay ^b^	*P. aeruginosa* Strain	Ref.
*Mytilus* spp.	Mussel	Myticalin A5 Myticalin A8 Myticalin C9 Myticalin D2	AMP	<8 µM 8 µM 8 µM 4 µM	MDA	ATCC27853	[102]
*Mytilus coruscus*	Mussel	Myticusin-beta	AMP	9.2 mm	URDA	KCTC1636	[103]
*Crassostrea gigas*	Pacific oyster	cgUbiquitin	AMP	0.6 µM	URDA	KCTC2004	[104]
*Echinus esculentus*	Sea urchin	EeCentrocin 1 EeCentrocin 2 EeStrongylocin 2	AMP	0.78 µM 0.78 µM 1.56 µM	MDA	ATCC27853	[105]
*Portunus pelagicus*	Crab	Crustin	AMP	50 µg/mL	Resazurin	HQ4006631	[106]
*Paralithodes camtschaticus*	King crab	Paralithocin 1-3	AMP	>100 µM	MDA	ATCC25853	[107]
*Scylla* *paramamosain*	Mud crab	Sphistin	AMP	24 µM	MDA	ATCC 9027	[108]
*Tachypleus* *tridentatus*	Horseshoe crab	TPAD	AMP	8 μg/mL 8–16 μg/mL	MDA	BAA 2108 ATCC 27853	[109]
*Katsuwonus pelamis*	Tuna	SHbAP	AMP	19 µg/mL	MDA	KCTC2004	[110]
*Epinephelus coioides*	Teleost fish	Epinecidin-1	AMP	50 µg/mL 3.12 µg/mL	MDA	ATCC 19660 R	[111]
*Oreochromis* *niloticus*	Teleost fish	TP-4	AMP	0.52 µg/mL	MDA	ATCC19660	[112]
*Oreochromis* *niloticus*	Teleost fish	Oreoch-1 Oreoch-2	AMP	35 µM 6.67 µM	MDA	NS*	[113]
*Hydrophis* *cyanocinctus*	Sea snake	Hc-CATH	AMP	5.17 µM	MDA	ATCC27853	[114]
*Myxine glutinosa L.*	Hagfish	Myxinidin	AMP	<10 µg/mL	MDA	Z61; K799	[115]
*Penicillium* *chrysogenum* DXY-1	Fungus	cyclo(L-Tyr-L-Pro)	AMP	6.2 mg/mL	MDA	PAO1	[116]
*Aspergillus clavatus* AS-107	Fungus	Cyclodepsipeptide	AMP	8.8 µM	MDA	NS*	[117]
*Bacillus subtilis*	Bacterium	Gageostatin A, B Gageostatin A+B	AMP	16 µg/mL 8 µg/mL	MDA	NS*	[118]
*Halobacillus litoralis* YS3016	Bacterium	Hololitaralin A Hololitaralin B	AMP	24 mm 17 mm	DDA	NS*	[119,120]
*Aplysia californica*	Sea hare	Escapin	Protein	0.31 µg/mL	MDA	PAO1	[121]
*Bacillus licheniformis* D1	Bacterium	BL-DZ1	Protein	3.12 µg/mL	MDA	PAO1	[122]
*Bacillus subtilis*	Bacterium	Gageotetrins A Gageotetrins B Gageotetrins C	Lipopeptides	0.06 µM 0.04 µM 0.02 µM	MDA	NS*	[123]
*Penicillium chrysogenum* QEN-24S	Fungus	Conidiogenone B	Diterpene	8 µg/mL	MDA	NS*	[124]
*Aspergillus* *insuetus* SD-512	Fungus	(5S,6S)-16,17-dihydroophiobolin H (6α)-21,21-O-dihydroophiobolin G Farnesylemefuranone D Farnesylemefuranone E Farnesylemefuranone F	Terpenoid	8 µg/mL 8 µg/mL 16 µg/mL 16 µg/mL 8 µg/mL	MDA	a QDIO-2	[125]
*Dictyoata acutiloba*	Seaweed	A1 C1	Terpenoid	0.9 µg/mL 0.89 µg/mL	MDA	MTCC741	[126]
*Sarcophyton* *trocheliophorum*	Soft coral	(5S)-3-[(3E,5S)-5-hydroxy-3-hepten-6-yn-1-yl]-5-methyl-2(5H)-furanone	Terpenoid	8 mm	DDA	NS*	[127]
*Axinella* *infundibuliformis*	Sponge	3β-Hydroxylup-20(29)-ene 3β-Hydroxylup-20(29)-en28-oic acid 3-oxo-lup-20(29)-en-28-oic acid	Terpenoid	24 mm 7 mm 10 mm	DDA	ATCC27853	[128]
*Streptomyces* *pratensis* NA-ZhouS1	Bacterium	Stremycin A-B	PK	16 µg/mL	MDA	NS*	[129]
*Penicillium* sp. RO-11	Fungus	Penipyranicins A Penipyranicins B Penipyranicins C Isopyrenulin	PK	18.4 µg/mL 5.2 µg/mL 1.4 µg/mL 4.7 µg/mL	MDA	NR-117678.1	[130]
*Streptomyces* sp. CMB-M0244	Bacterium	Mollemycin A	PK	IC_50_ 50 nM	MDA	ATCC 27853	[131]
*Penicillium* sp. BB1122	Fungus	Neocitreoviridin Penicillstressol Isopenicillstressol 10Z-isocitreoviridinol	PK	4 µg/mL 4 µg/mL 4 µg/mL 8 µg/mL	MDA	CMCC(B)10104	[132]
*Streptomyces* sp. HB202	Bacterium	Mayamycin	PK	IC_50_ 2.5 µg/mL	MDA	DSM 50071	[133]
*Penicillium* sp. HDN151272	Fungus	Ketidocillinone B Ketidocillinone C	PK	1.56 mg/mL 6.25 mg/mL	MDA	NS*	[134]
*Shewanella algae* MTCC 12715	Bacterium	14-(14b,14c-dimethylbutyl)-12-methoxy-18-oxo-11,15-dioxacyclododecan-8-yl 1-((50-hydroxyfuran-10-yl)oxy)benzoate;	PK	21 mm–1.5 µg/mL	DDA; MDA	ATCC27853	[135]
		14-(sec-butyl)-12-methoxy-12-methyl-18-oxo-11,15-dioxacyclododecan-8-yl1-((50-hydroxyfuran-10-yl)oxy)benzoate		24 mm–3 µg/mL			
*Bacillus* *Amyloliquefacien* MTCC 12716	Bacterium	Methyl 1′-((2E,4E,14E)-9,12-dihydroxy-15-isopropyl-1,6- dioxohexadecahydro [1]oxacyclononadecino[3,4-f]isobenzofuranyl) benzoate;	PK	3.12 µg/mL	MDA	ATCC27853	[136]
		E)-Ethyl 15-ethyl-9,12-dihydroxy-25-(2-hydroxy-3-(methoxycarbonyl)phenyl)-1-oxo-octadecahydro-1 H -furopyrano[2,3- c]oxacyclononadecine-6-carboxylate;		0.75 µg/mL			
		((E)-Ethyl 15-ethyl-12-hydroxy-25-(2 -hydroxy-3-(methoxycarbonyl)phenyl)-24-methyl-1-oxo-icosahydro-1 H-furopyrano[2,3-c]oxacyclononadecine-6-carboxylate		1.50 µg/mL			
*Bacillus* *amyloliquefacien* MTCC 12713	Bacterium	4,27,39-Trihydroxy-7,8,10,11,16,17,25,26,27,28-decahydro-37-methyl-macrobrevin;	PK	3.12 µg/mL–19 mm	MDA, DDA	ATCC27853	[137]
		7,8,16,17,25,26-Hexahydro-macrobrevin;		6.25 µg/mL–13 mm			
		7,8,16,17,25,26-Hexahydro-41-hydroxy-macrobrevin-31-acetate;		1.56 µg/mL–23 mm			
		7,8,16,17,25,26-Hexahydro-28-nor-methyl-5-methoxy-macrobrevin		3.12 µg/mL–22mm			
*Bacillus* *amyloliquefacien* MTCC 12713	Bacterium	18,19-Dihydro-6-hydroxy-8-propyl carboxylate difficidin	PK	0.006 µM–17 mm	MDA, DDA	ATCC27853	[138]
		5-Ethoxy-28-methyl-(9-methyl-19-propyl dicarboxylate) difficidin		0.004 µM–26 mm			
		(6-Methyl-9-propyl dicarboxylate)-19-propanone difficidin		0.002 µM–23 mm			
		20-Acetyl-(6-methyl-9-isopentyl dicarboxylate) difficidin		0.002 µM-25 mm			
*Aspergillus niger* ASSB4	Fungus	RF-3192C	PK	15 mm	DDA	ATCC27853	[139]
*Microsphaeropsis* sp. RA10-14	Fungus	Microketides A Microketides B	PK	0.19 µg/mL 1.56 µg/mL	MDA	NS*	[140]
*Actinoalloteichus* *cyanogriseus* WH1-2216	Bacterium	Caerulomycin A Caerulomycin C	Alkaloid	21.8 µg/mL 38.6 µg/mL	ADA	NS*	[141]
*Clathria cervicornis*	Sponge	Crambescidin 800	Alkaloid	1 µg/mL	MDA	ATCC 10145	[142]
*Agelas dilatata*	Sponge	Bromoageliferin	Alkaloid	32 µg/mL 8 µg/mL	MDA	PAO1 ATCC 27853	[143]
*Pachychalina* sp.	Sponge	Cyclostellettamine C	Alkaloid	8.6 µg/mL 18.8 µg/mL	MDA	ATCC27853 Pa13, PaP1	[144]
		Cyclostellettamine E		18.8 µg/mL 18.8 µg/mL 9.4 µg/mL		ATCC27853, PaP1 Pa13	
		Cyclostellettamine F		4.7 µg/mL 9.4 µg/mL		PaP1 Pa13	
*Callyspongia* *siphonella*	Sponge	5-bromo trisindoline 6-bromo trisindoline	Alkaloid	256 µg/mL 256 µg/mL	MDA	PAO1	[145]
*Dendrilla nigra*	Sponge	Denigrins A Denigrins B Denigrins C	Alkaloid	100 µg/mL 25 µg/Ml 12.5 µg/mL	MDA	ATCC 27853	[146]
*Tegella* cf. *spitzbergensis*	Bryozoan	Ent-eusynstyelamide B Eusynstyelamide D Eusynstyelamide E Eusynstyelamide F	Alkaloid	25 µg/mL 25 µg/mL 25 µg/mL 12.5 µg/mL	MDA	ATCC 27853	[147]
*Acanthophora* *spicifera*	Sponge	Velutin	Flavone	26.8 mm	DDA	ATCC9027	[148]
*Siphonochalina* *siphonella*	Sponge	Siphonocholin	Steroid	64 µg/mL	MDA	PAO1	[149]
*Dysidea granulosa*	Sponge	2-(20,40-dibromophenoxy)-3,5-dibromophenol	Diphenylether derivative	4 µg/mL	MDA	NS*	[150]
*Aspergillus versicolor* OUCMDZ-2738	Fungus	brevianamide K Diorcinol C Diorcinol E Diorcinol J Diorcinol Methyl diorcinol-4-carboxylate	Diphenylether derivative	IC_50_ 92.2 µM IC_50_ 46.2 µM IC_50_ 101.9 µM IC_50_ 50.9 µM IC_50_ 17.4 µM IC_50_ 13.9 µM	MDA	ATCC10145	[151]
*Ecteinascidia* *turbinate*	Ascidian	Ecteinamycin	Polyether	8.0 μg/mL	MDA	ATCC 27853	[152]
*Bacillus subtilis*	Bacterium	DEHP	Phthalate derivative	8 µg/mL	MDA	ATCC 9027	[153]
*Bacillus licheniformis* 09IDYM23	Bacterium	Ieodoglucomide C Ieodoglycolipid	Glycolipid	0.01 µM 0.03 µM	MDA	NS*	[154]
*Bacillus* sp. REB264	Bacterium	PAGI264	NS	15 µg/mL	MDA	NS*	[155]
*Bacillus* sp.	Bacterium	Macrolactins A1 Macrolactins B1	Macrolide	0.055 µM 0.055 µM	MDA	NS*	[156]
*Bacillus subtilis*	Bacterium	Gageomacrolactins 1 Gageomacrolactins 2 Gageomacrolactins 3	Macrolide	0.03 µM 0.05 µM 0.05 µM	MDA	NS*	[157]
*B. amyloliquefaciens* MTCC 12716	Bacterium	Bacvalactones 1 Bacvalactones 2 Bacvalactones 3	Macrolide	3.12 µg/mL 3.00 µg/mL 1.5 µg/mL	MDA	ATCC 27853	[158]
*Bacillus subtilis* MTCC 10403	Bacterium	7-O-6′-(2″-acetylphenyl)-5′-hydroxyhexanoate-macrolactin	Macrolide	12.5 µg/mL	MDA	MTCC 429	[159]

^a^ AMP: antimicrobial peptides; PK: polyketide; ^b^ MDA: microdilution assay; URDA: radial diffusion assay; DDA: disk diffusion assay; *NS: not specified.

**Table 2:** List of marine anti-virulence compounds against *P. aeruginosa*.

Producer Organism	Type of Organism	Compound	Class	Activity	Assay	*P. aeruginosa* Strain	Ref.
*Pseudoalteromonas* sp. 1400	Bacterium	AlyP1400	Protein	Antibiofilm	Biofilm disruption	CF27 PA14	[160,161]
*Pseudoalteromonas* sp. 129-1	Bacterium	Protease	Protein	Antibiofilm	Biofilm inhibition	PAO1	[162]
*Pseudoalteromonas* sp. 3j6	Bacterium	Alterocin	Protein	Antibiofilm	Biofilm inhibition	PAO1	[163]
*Bacillus velezensis* DH82	Bacterium	YtnP	Protein	Anti-QS Antibiofilm	Inhibition of virulence factors Biofilm inhibition	PAO1	[164]
*Psychrobacter* sp. M9-54-1	Bacterium	AHL-Acylase	Protein	Anti-QS	QS genes downregulation	PAO1	[165]
*Nesterenkonia* sp. MSA31	Bacterium	Nesfactin	Lipopeptide	Anti-QS Antibiofilm	Inhibition of virulence factors Biofilm disruption	PAO1 FSPA02	[166]
*Penicillium chrysogenum* DXY-1	Fungus	cyclo(L-Tyr-L-Pro)	Dipeptide	Annt-QS Antibiofilm	*las* and *rhl* reduced expression Biofilm inhibition	PAO1	[116]
*Penicillium chrysogenum* DXY-1	Fungus	Tyrosol	Dipeptide	Anti-QS Antibiofilm	Inhibition of virulence factors Biofilm inhibition	PAO1	[167]
*Rheinheimera aquimaris* QSI02	Bacterium	Cyclo(Trp-Ser)	Dipeptide	Anti-QS Antibiofilm	Inhibition of virulence factors Biofilm inhibition	PAO1	[168]
*Pestalotiopsis sydowiana* PPR	Fungus	Cyclo(Leu-Pro)	Dipeptide	Anti-QS	Inhibition of virulence factors	PAO1	[169]
*Cladosporium* sp. Z148	Fungus	Cladodionen	PK	Anti-QS	Inhibition of virulence factors	PAO1	[170]
*Fusarium* sp. Z10	Fungus	Equisetin	PK	Anti-QS Antibiofilm	Inhibition of virulence factors Biofilm inhibition	PAO1	[171]
*Agelas dilatata*	Sponge	Bromoageliferin	Alkaloid	Antibiofilm Anti-virulence	Biofilm inhibition *G. mellonella* survival assay	PAO1 ATCC 27853	[143]
*Callyspongia siphonella*	Sponge	5-bromo trisindoline 6-bromo trisindoline	Alkaloid	Antibiofilm	Biofilm inhibition	PAO1	[145]
*Oceanobacillus* sp. XC22919	Bacterium	2-methyl-N-(2′-phenylethyl) butyramide; 3-methyl-N-(2′-phenylethyl)-butyramide	Alkaloid	Anti-QS Antibiofilm	Inhibition of virulence factors Biofilm inhibition	PAO1	[172]
*Halodule pinifolia*	Seagrass	4-methoxybeanzoic acid (4-MBA)	Benzoic acid derivative	Anti-QS Antibiofilm	Inhibition of virulence factors Biofilm inhibition	PAO1	[173]
*Pestalotiopsis sydowiana* PPR	Fungus	4-Hydroxyphenylacetamide	phenylacetic acid derivative	Anti-QS	Inhibition of virulence factors	PAO1	[169]
*Vibrio alginolyticus*	Bacterium	Tyramine N-acetyltyramine	Amine	Anti-QS	Inhibition of virulence factors	PAO1	[165]
*Siphonochalina siphonella*	Sponge	Syph-1	Steroid	Anti-QS Antibiofilm	Inhibition of virulence factors Biofilm inhibition	PAO1	[149]
*Oceanobacillus* sp. XC22919	Bacterium	Benzyl benzoate	Benzoic acid derivative	Anti-QS Antibiofilm	Inhibition of virulence factors Biofilm inhibition	PAO1	[172]
*Streptomyces* sp.	Bacterium	5-octylfuran-2(5H)-one	Lactone butenolide	Antibiofilm	Biofilm degradation Biofilm disruption	PAO1	[174]
*Staphylococcus hominis*	Bacterium	DL-homocysteine thiolacton	Lactone	Anti-QS Antibiofilm	QS genes downregulation Biofilm inhibition	PAO1	[175]
*Staphylococcus saprophyticus* SBPS-15	Bacterium	Staphylosan	Glycolipid	Antibiofilm	Biofilm degradation Biofilm disruption	BHKH	[176].
*Serratia marcescens*	Bacterium	-	Glycolipid	Antibiofilm	Attachment inhibition Biofilm degradation Biofilm disruption	PAO1	[177]
*Lyngbya majuscola*	Cyanobacterium	Lyngbyoic acid	Fatty Acid	Anti-QS	Inhibition of virulence factors	PAO1	[178]

## Data Availability

Not applicable.
